# Risk stratification by ultrasound and mammography for screen-detected non-palpable breast cancer in Chinese women

**DOI:** 10.3389/fonc.2025.1555743

**Published:** 2025-10-17

**Authors:** Ying Xu, Ru Yao, Yan Lin, Feng Mao, Xiaohui Zhang, Songjie Shen, Bo Pan, Yidong Zhou, Qiang Sun

**Affiliations:** Department of Breast Surgery, Peking Union Medical College Hospital, Chinese Academy of Medical Sciences and Peking Union Medical College, Beijing, China

**Keywords:** Chinese women, non-palpable breast cancer, ultrasound, mammography, prognosis

## Abstract

**Background:**

Mammography (MG) and ultrasound (US) are currently the ‘real-world’ initial imaging tests for breast cancer in China. Previously, we demonstrated that US and MG detected non-palpable breast cancer (NPBC) had similar survival. This study was performed to validate the hypothesis whether MG+/US- NPBC could be taken as ultra-low risk cancer.

**Method:**

From 2015-2018, 3,113 consecutive patients received biopsy with initial positive screening. Among them, 2,591 US positive patients underwent US-guided biopsy. Meanwhile, 371 MG+/US- patients underwent MG-guided biopsy. Clinical characteristics, treatment and 5-year disease free survival (DFS) and overall survival (OS) were analyzed. Prognostic factors of NPBC were identified.

**Results:**

We identified 419 cases of US+/MG-, 225 cases of US+/MG+, and 118 cases of US-/MG+ breast cancers, yielding positive predictive values (PPVs) of 21.6%, 34.7%, and 22.6%, respectively. Notably, among NPBC with US-/MG+ features, a significantly higher proportion exhibited DCIS (50.8%, P<0.001), multifocality (18.5%, P = 0.003), underwent breast-conserving surgeries (66.1%, P<0.001), and did not receive chemotherapy or radiotherapy (64.4% & 66.9%, P<0.001 & P = 0.032). MG+/US- patients demonstrated improved DFS compared to US+/AnyMG (P = 0.035), with no significant difference in OS (P = 0.48). Univariate and multivariate Cox regression analysis identified age, TNM stage, lymphovascular invasion (LVI), and estrogen receptor (ER) status as significant DFS predictors(P<0.05), with ER status alone being significant for OS (P = 0.002).

**Conclusion:**

MG^+^/US^-^ NPBC was associated with a favorable prognosis in this study, potentially representing an “ultra-low-risk” subtype of breast cancer that warrants further investigation. Hence US had the potential of stratifying the screen-detected NPBC into regular low risk (US+/MG+ and US+/MG-) and ultra-low risk (MG+/US-).

## 1 Introduction

The pivotal publications of the Greater New York Health Insurance Plan (HIP) trial (1) and the Swedish Two-County Trial (TCT) (2) in 1985 established mammography (MG) as a cornerstone of breast cancer screening in Western countries. Subsequent two decades saw widespread MG adoption, accompanied by a rise in stage I or *in-situ* breast cancers detection, without a proportional decline in advanced-stage (II-IV) incidence or in breast cancer mortality (3–6). This suggests that MG-detected cancers may often be inherently low-risk. Moreover, studies indicate that MG exhibits greater sensitivity towards Luminal A subtype breast cancers, as opposed to the more aggressive Basal-like subtype (7, 8).

Given the unique anatomical characteristics of Chinese women, who tend to have smaller and denser breasts, ultrasound (US) has emerged as a general diagnostic tool for breast cancer. Our previous multi-center randomized controlled trial groundbreakingly demonstrated that US significantly enhances the sensitivity and accuracy of breast cancer detection (9). Building upon this, we further validated in a hospital-screening setting that US and MG yield comparable and similar survival outcomes for non-palpable breast cancers (NPBC) (10). This aligns with Western practices, where US complements MG in dense breasts (11, 12). Notably, the ACRIN 6666 study underscored the distinct strengths of each modality, with US detecting a higher proportion of invasive and node-negative cancers, while MG identified more ductal carcinoma *in situ* (DCIS) cases (P < 0.001) (13).

In conducting combined US and MG screening, prior research has consistently demonstrated that MG+/US- microcalcifications are typically non-indicative of malignant tumors, with a benignity rate ranging from 65% to 90% (14, 15). Nevertheless, the presence of MG+/US- microcalcifications can occasionally result in unwarranted biopsy procedures, which not only escalate medical costs for patients but also potentially elevate their future risk of breast cancer (16).

We had proposed a hypothesis that asymptomatic MG+/US− micro-calcification might not necessitate immediate invasive interventions and could be safely monitored until they become US-positive. In short, we propose a “watch-and-wait” approach for such asymptomatic MG+/US− micro-calcifications, with biopsy reserved for when they transition to US + (17). If this hypothesis is to be applied in clinical practice, careful consideration must be given to ethical and clinical acceptability. This would include obtaining approval from an ethics committee, ensuring fully informed patient consent, establishing a rigorous follow-up monitoring system, developing clear indications for surgical intervention, and assessing potential psychosocial impacts. To validate this hypothesis, we conducted a study comparing the clinicopathological features and survival outcomes among Chinese women with non-palpable breast cancers (NPBC) categorized as MG+US+, MG+US−, and MG−US+. Our objective was to ascertain whether MG^+^/US^-^ non-palpable breast cancer (NPBC) can be classified as an ultra-low-risk subtype—specifically referring to a tumor subgroup characterized by minimal risk of disease progression, excellent long-term prognosis, and potential eligibility for reduced therapeutic intervention. A clear definition of this concept is critical for developing individualized treatment strategies.

## 2 Materials and methods

### 2.1 Patients selection

From January 2015 to December 2018, PUMCH’s breast clinics conducted diagnostic ultrasound (US) and mammography (MG) on 5,040 asymptomatic women who underwent opportunistic breast cancer (BC) screening, through self-presentation. Patients were selected according to the following exclusion criteria: 1) history of prior breast malignancy; 2) unavailability of mammography (MG) or ultrasound (US) results; 3) both MG and US assessments classified as negative (BI-RADS category 0–3); 4) absence of biopsy or surgical treatment. After applying these exclusions, a cohort of 3,113 asymptomatic patients with positive imaging findings on US or MG (classified as BI-RADS 4a, 4b, 4c, or 5) were identified. Among them, 1944 individuals showed positive US results with negative MG outcomes, whereas 522 patients presented with positive MG results and negative US findings. Notably, 647 patients demonstrated concurrent positivity on both modalities. All patients participating in this study were asymptomatic, with their masses or calcifications detected either serendipitously during routine health examinations or as a part of a dedicated cancer screening program.

### 2.2 Screening, biopsy and follow-up procedure

All the patients underwent both mammography and ultrasound before surgery. A standard two-view mammography was performed by using digital mammography. Screening ultrasound was performed by using color Doppler and high-resolution transducers. The Breast Imaging- Reporting and Data System (BI-RADS) lexicon was used to define lesions. Referring to BI-RADS categories: 1, negative; 2, benign; 3, probably benign; 4, suspicious malignancy; and 5, highly suggestive of malignancy. Lesions with BI-RADS categories 4 and 5 considered as “screening positive”.

2591 US positive patients underwent US-guided open surgical biopsy, regardless of the result of mammography. Meanwhile, 371 MG positive/US negative patients underwent MG-guided open surgical biopsy. All procedures were consistently performed by the same breast surgery team to minimize variability. 419 US+MG- NPBC, 225 US+MG+ NPBC and 118 US-MG+ NPBC were diagnosed.

Patients lacking either breast ultrasound or mammography results were excluded from the study. Those with unknown postoperative pathological indicators were documented as “Unknown.” All enrolled patients underwent regular follow-up according to a standardized protocol established by our institutional Breast Cancer Center to ensure consistency in monitoring intervals and evaluation methods. Follow-up commenced at the time of diagnosis and consisted of clinical physical examinations and breast ultrasound every six months for the first three years, followed by annual evaluations thereafter. Follow-up data were primarily collected through systematic review of electronic medical records from outpatient visits and were cross-verified by two independent researchers to ensure accuracy. For patients who did not return for scheduled visits, follow-up was conducted via telephone contact, and they were advised to undergo required examinations at local tertiary Grade A hospitals. All external imaging reports were re-evaluated by two senior radiologists from our hospital to maintain consistency in imaging assessment. Patients lost to follow-up were censored in the data analysis. Survival status (alive or deceased) and recurrence events (local recurrence or distant metastasis) were strictly determined based on clinical, imaging, and pathological evidence. Disease-free survival was defined as the time from surgery to the first occurrence of any recurrence (local/regional recurrence or distant metastasis) or death from any cause. Overall survival was defined as the time from surgery to death from any cause.

### 2.3 Statistical analysis

The clinicopathological features were evaluated through rigorous statistical analyses, employing the t-test, chi-square test, and Kruskal-Wallis test. Specifically, the independent samples t-test was utilized for comparative assessment of measurement data, the Pearson Chi-square test was applied to compare categorical count data, and the Kruskal-Wallis test was selected for grading information analysis. The Kaplan-Meier curve methodology was deployed to meticulously analyze and compare survival outcomes, inclusive of 5-year predicted disease-free survival (DFS) and overall survival (OS). Both Cox univariate and multivariate analyses were systematically conducted to pinpoint prognostic factors that significantly impact DFS and OS. A p-value < 0.05 was considered statistically significant for single comparisons. For analyses involving multiple comparisons, the Bonferroni correction was applied, and a corrected p-value < 0.0167 was deemed statistically significant. The software package R version 4.2.2 was used for all of the statistical analyses.

## 3 Results

### 3.1 Descriptive information

Between 2015 and 2018, a cohort of 3,113 consecutive patients underwent either ultrasound (US)-guided or mammography (MG)-guided biopsy at Peking Union Medical College Hospital. Subsequent analysis of medical records identified 2,591 patients within this cohort who had undergone biopsy based on initial positive US screening results (BI-RADS 4 and 5), yielding diagnoses of 419 US-positive/MG-negative (US+/MG-) and 225 US-positive/MG-positive (US+/MG+) non-palpable breast cancers (NPBC). Meanwhile, among the 371 patients with initially negative US screening results (BI-RADS 1, 2, or 3) but positive MG findings (BI-RADS 4 or 5), MG-guided biopsies resulted in the detection of 118 MG-positive/US-negative (MG+/US-) NPBC cases. The patient selection methodology is illustrated in **Supplementary Figure 1**.

**Figure 1:**
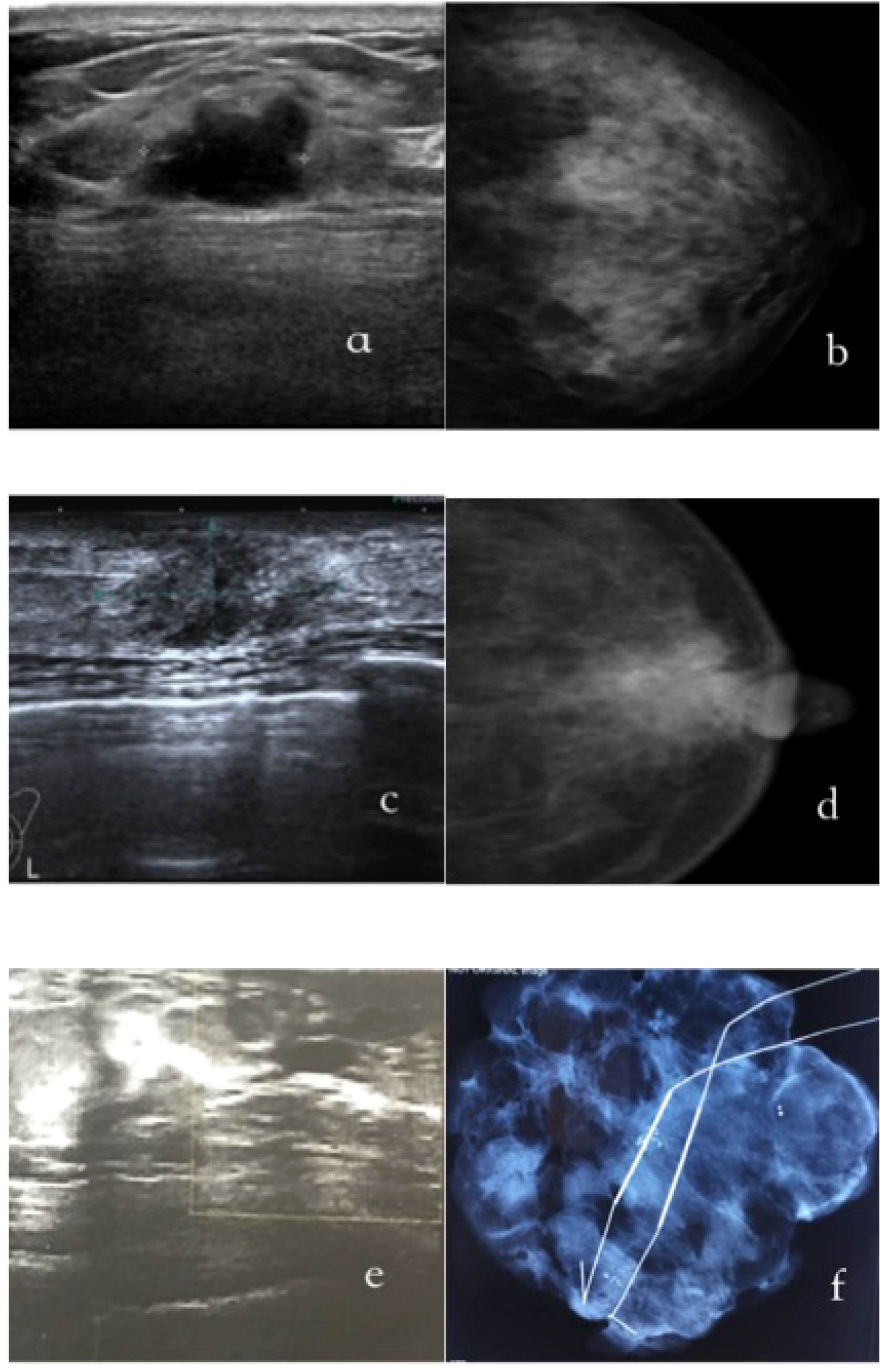
The typical images of US+/MG-(a/b), US+/MG-(c/d) and US-/MG+(e/f) NPBC breast cancer. **(A, B)** were images of a 35-year-old woman with a T1cN0M0, I stage;Luminal B NPBC; **(C, D)** were images of a 55-year-old woman with a TisN0M0, 0 stage NPBC; **(E, F)** were images of a 50-year-old woman with a multifocal TisN0M0, 0 stage NPBC.

**Figure 1** exhibits representative imaging findings for three distinct patient groups. Specifically, **Figures 1A, B** display ultrasound and mammography images of a 35-year-old female patient diagnosed with non-palpable breast cancer (NPBC) and classified as US+MG-. The ultrasound images reveal irregular hypoechoic nodules, indicative of a heightened malignancy risk, whereas mammography shows negative finding. **Figures 1C, D** showcase a 55-year-old female patient with a suspected malignant breast mass, where nodule and calcifications are detected on both ultrasound and mammography, reinforcing the suspicion of malignancy. Lastly, **Figures 1E, F** depict a 50-year-old female patient classified as US-MG+, where the ultrasound images are devoid of malignant lesions, yet clustered calcifications are evident on molybdenum target imaging. The diagnosis of breast cancer in this case was definitively established through molybdenum target-guided biopsy pathology.

The positive predictive values (PPVs) for these three groups (US+/MG-, US+/MG+ and US-/MG+) were calculated as 21.6%, 34.7%, and 22.6%, respectively. Further analysis revealed that US was more adept at detecting invasive cancers, whereas MG detected a higher proportion of ductal carcinoma *in situ* (DCIS). Specifically, in the US+/MG+ NPBC group, invasive cancers comprised 86.2% of diagnoses, with a similar percentage (81.3%) observed in the US+/MG- group. In contrast, only 48.3% of cases in the MG+/US-NPBC group were invasive, with over 50% of patients exhibiting intraductal cancer. *Post-hoc* pairwise comparisons with Bonferroni correction (adjusted α = 0.0167) revealed that the PPV in the US+/MG+ group was significantly higher than that in the US+/MG- group (34.7% vs. 21.6%, p* = 0.001) and the MG+/US- group (34.7% vs. 22.6%, p* = 0.002). No significant difference was found between the US+/MG- and MG+/US- groups (p* = 0.685). (Refer to **Table 1** for details).

**Table 1:** Comparison of positive predictive value (PPV), pathology and prognosis of US+/MG-, US+/MG+ and MG+/US- NPBC.

Pathology\radiology (2015-2018)	US-detected NPBL (N = 2,591)		MG-detected NPBL (N = 522)
MG & US positivity	US+/MG- (N = 1,944)	US+/MG+ (N = 647)	MG+/US- (N = 522)
Imaging presentation	Nodule	Nodule + micro-calcifications	Micro-calcifications
Breast cancer (PPV %)*	419 (21.6%)	225 (34.7%)	118 (22.6%)
Pathology (p<0.001) DCIS (%) Invasive (%)	58 (13.8) 361 (86.2)	42 (18.7) 183 (81.3)	61 (51.7) 57 (48.3)
5-Year survival DFS (%,95%CI) OS (%,95%CI)	90.9(88.2-93.7) 96.9(95.3-98.6)	90.7(86.9-94.5) 98.2(96.5-100)	96.6(93.4-99.9) 98.3(96.0-100)

*p values for *post-hoc* pairwise comparisons of PPV are adjusted using the Bonferroni method.

The median follow-up time for the study cohort was 76 months, ranging from 41 to 110 months. Notably, 12 patients were lost to follow-up, and among the remaining participants, 64 experienced recurrence or metastasis. Specifically, local recurrence was observed in 13 patients, while 13 patients suffered from lung metastasis. Additionally, bone metastasis was identified in 15 patients, and liver metastasis occurred in 5 patients. Furthermore, 10 patients exhibited multiple metastases involving two or more organs, and another 8 patients developed cervical lymph node metastasis. Unfortunately, 34 patients passed away during the follow-up period, with 27 deaths attributed to breast cancer and 7 deaths resulting from non-breast cancer-related causes. (**Supplementary Table 1**).

### 3.2 Clinicopathological characteristics

**Table 2** comprehensively outlines the clinical and pathological profiles of three distinct cohorts of non-palpable breast cancer (NPBC) patients. Notably, no statistically significant disparities were observed among these three groups in age at diagnosis, different age groups, lymph node status, histological grade, bilateral breast cancer occurrence, LVI status, hormone receptor status, Ki-67 index, or the receipt of endocrine and targeted therapies. (P>0.05).

**Table 2:** Clinicopathologic characteristics of screening-detected NPBC from hospital-based population.

Characteristics	No. (%) of patients			P^a^
	US+/MG-	US+/MG+	US-/MG+	
Total	419	225	118	
Age(years)				
Mean± SD	51.26 ± 11.66	52.42 ± 11.01	49.21 ± 9.48	0.081
Age at diagnosis				0.156*
<40	65 (15.5)	28 (12.4)	19 (16.1)	
40-49	136 (32.5)	78 (34.7)	46 (39.0)	
50-59	115 (27.4)	62 (27.6)	35 (29.7)	
≥60	103 (24.6)	57 (25.3)	18 (15.3)	
pT simple				<0.001*
T0	56 (13.4)	40 (17.8)	60 (50.8)	
T1	326 (77.8)	174 (77.3)	53 (44.9)	
T2	37 (8.8)	11 (4.9)	5 (4.2)	
pT				<0.001*
Tis	56 (13.4)	40 (17.8)	60 (50.8)	
T1a	55 (13.1)	20 (8.9)	17 (14.4)	
T1b	112 (26.7)	58 (25.8)	12 (10.2)	
T1c	159 (37.9)	96 (42.7)	24 (20.3)	
T2	37 (8.8)	11 (4.9)	5 (4.2)	
Lymph node status				0.249
Negative	356 (85.0)	180 (80.0)	100 (84.7)	
Positive	63 (15.0)	45 (20.0)	18 (15.3)	
Number of positive LN				0.484
Mean± SD	0.35 ± 1.51	0.48 ± 1.91	0.87 ± 3.69	
pN				0.249*
N0	356 (85.0)	180 (80.0)	100 (84.7)	
N1	51 (12.2)	34 (15.1)	11 (9.3)	
N2	6 (1.4)	8 (3.6)	3 (2.5)	
N3	6 (1.4)	3 (3.4)	4 (3.4)	
TNM stage^b^				<0.001*
0	56 (13.4)	40 (17.8)	60 (50.8)	
I	273 (65.2)	134 (59.6)	38 (32.2)	
IIa	69 (16.5)	37 (16.4)	13 (11.0)	
IIb	9 (2.1)	3 (1.3)	0 (0.0)	
IIIa	6 (1.4)	8 (3.6)	3 (2.5)	
IIIc	6 (1.4)	3 (1.3)	4 (3.4)	
Histological grade				0.139*
Low grade	88 (21.0)	35 (15.6)	33 (28.0)	
Medium grade	234 (56.0)	126 (56.0)	50 (42.4)	
High grade	97 (23.2)	64 (29.7)	35 (29.7)	
Focality				0.003
Monofocal	384 (91.6)	206 (91.6)	96 (81.4)	
Multifocal	35 (8.4)	19 (8.4)	22 (18.6)	
Laterality				0.717
Unilateral	400 (95.5)	216 (96.0)	111 (94.1)	
Bilateral	19 (4.5)	9 (4.0)	7 (5.9)	
LVI				0.479
No	404 (96.4)	214 (95.1)	111 (94.1)	
Yes	15 (3.6)	11 (4.9)	7 (5.9)	
ER				0.952
Negative	59 (14.1)	30 (13.3)	17 (14.4)	
Positive	360 (85.9)	195 (86.7)	101 (85.6)	
PR				0.738
Negative	84 (20.0)	51 (22.7)	25 (21.2)	
Positive	335 (80.0)	174 (77.3)	93 (78.8)	
Hormone receptor				0.818
Negative	59 (14.1)	28 (12.4)	17 (14.4)	
Positive	360 (85.9)	197 (87.6)	101 (85.6)	
Her2 status^				0.016
Negative	342 (81.6)	162 (72.0)	91 (77.1)	
Positive	66 (15.8)	55 (24.4)	27 (22.9)	
Unknown	11 (2.6)	8 (3.6)	0 (0)	
Ki67				0.365
<14%	194 (46.3)	95 (42.2)	47 (39.8)	
≥14%	225 (53.7)	130 (57.8)	71 (60.2)	
Pathological Subtype^c^				<0.001*
DCIS	58 (13.8)	42 (18.7)	61 (51.7)	
Luminal A	144 (34.4)	62 (27.6)	15 (12.7)	
Luminal B	165 (39.4)	103 (45.8)	31 (26.3)	
Her-2	16 (3.8)	8 (3.6)	5 (4.2)	
TNBC	36 (8.6)	10 (4.4)	6 (5.1)	
Surgery				<0.001
Mastectomy	244 (58.2)	111 (49.3)	40 (33.9)	
Breast conserving	175 (41.8)	114 (31.1)	78 (66.1)	
Chemotherapy				0.032
No	226 (53.9)	121 (53.8)	79 (66.9)	
Yes	193 (46.1)	104 (46.2)	39 (33.1)	
Radiotherapy				<0.001
No	176 (42.0)	114 (50.7)	76 (64.4)	
Yes	243 (58.0)	111 (49.3)	42 (35.6)	
Anti-HER2 therapy				0.137
No	372 (88.8)	188 (83.6)	105 (89.0)	
Yes	47 (11.2)	37 (16.4)	13 (11.0)	
Endocrine therapy				0.916
No	52 (12.4)	27 (12.0)	16 (13.6)	
Yes	367 (87.6)	198 (88.0)	102 (88.6)	

a Bold type indicates statistical significance.

b TNM stage is according to the 8^th^ AJCC cancer staging system.

c Immunophenotype of invasive NPBC is according to the immunohistochemical subtype of 2013 St. Gallen Consensus.

^*^The comparison was performed by Kruskal Wallis test.

Significant variations in tumor size and TNM staging were discernible among the three patient groups. The US-/MG+ group demonstrated a higher proportion of DCIS, exceeding 50%, in contrast to the other two groups where the proportion of DCIS remained below 20%. Additionally, the US -/MG+ group stood out with a statistically significant increased incidence of multifocal breast cancer, accounting for 18.6% of cases compared to the other groups.

Regarding HER2 status, distinct differences emerged among the three groups. Especially, the US+/MG- NPBC group exhibited a higher prevalence of HER2-negative breast cancers (81%), while the HER2 profiles of the remaining two groups were comparable (72.0% and 77.1%).

With respect to pathological subtype, the three patient groups also displayed significant differences. Specifically, the US+/MG- group had a higher proportion of triple-negative breast cancers (TNBC), reaching 8.6%, compared to the other two groups. Conversely, the US+/MG+ cohort was characterized by a higher prevalence of Luminal B breast cancers, comprising 45.8% of cases.

Regarding surgical approaches, the US-/MG+ group exhibited a notably elevated percentage of patients undergoing breast-conserving surgery, reaching 66%. Conversely, the US+/MG+ and US+/MG- groups had lower proportions of such patients, with 41.8% and 31.1%, respectively. Given the heightened prevalence of DCIS within the US-/MG+NPBC cohort, it is understandable that the corresponding rates of adjuvant chemotherapy and radiotherapy administration are comparatively lower across the three groups (P<0.05).

### 3.3 Survival outcomes and prognostic factors

**Figure 2:**
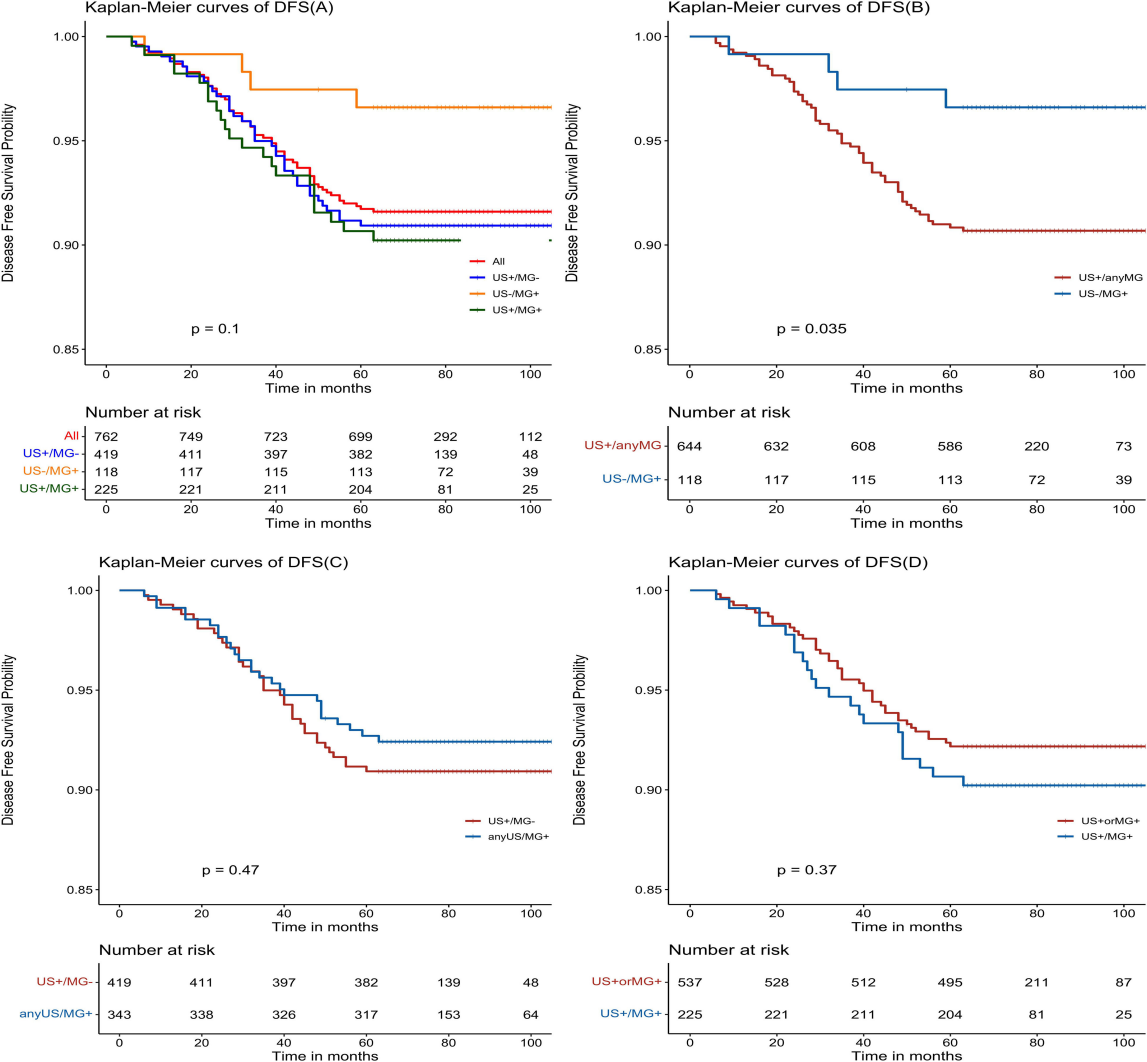
Kaplan-Meier estimated prognosis of NPBC patients. **(A)** There was no significant difference in DFS among US+/MG-, US+/MG+ and US-/MG+ NPBC. (P = 0.10). **(B)** There was significant difference in DFS between US+/any MG and MG+/US- NPBC(P = 0.035). **(C)** There was no significant difference in DFS between MG+/any US and US+/MG- NPBC(P = 0.47). **(D)** There was no significant difference in DFS between US+ or MG+ and US+/MG+ NPBC. (P = 0.37).

**Figure 3:**
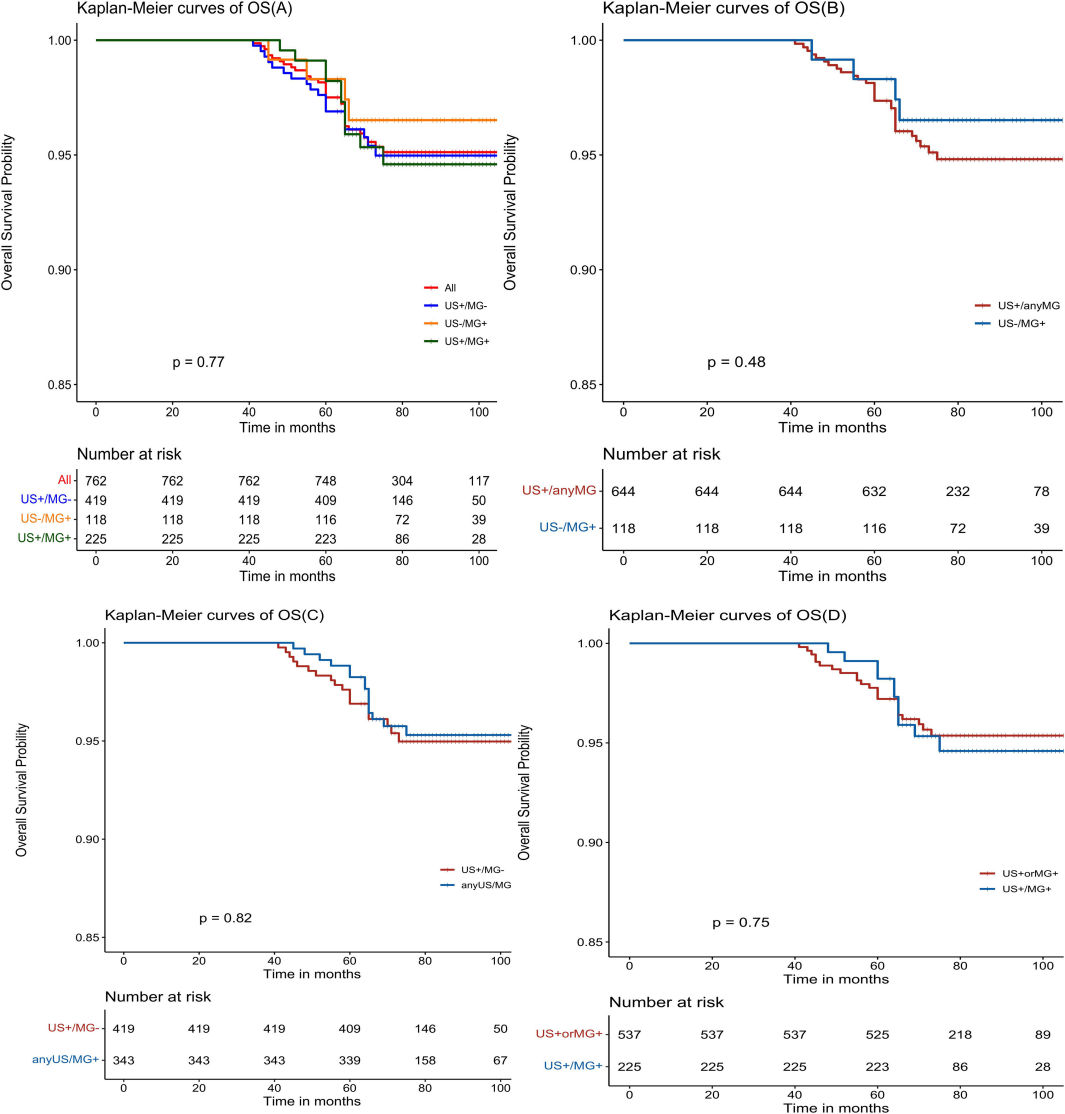
Kaplan-Meier estimated prognosis of NPBC patients. **(A)** There was no significant difference in OS among US+/MG-, US+/MG+ and US-/MG+ NPBC(P = 0.77). **(B)** There was no significant difference in OS between US+/any MG and MG+/US- NPBC(P = 0.48). **(C)** There was no significant difference in DFS between MG+/any US and US+/MG- NPBC(P = 0.82). **(D)** There was no significant difference in DFS between US+ or MG+ and US+/MG+ NPBC (P = 0.75).

The Kaplan-Meier estimates reveal favorable outcomes for all NPBC patients, with 5-year disease-free survival (DFS) and overall survival (OS) of 91.7% (95% CI: 89.8%-93.7%, **Figure 2A**) and 97.5% (95% CI: 96.4%-98.6%, **Figure 3A**), respectively. When examining specific subgroups—US+/MG+, US+/MG+, and US-MG+—the 5-year DFS were 90.9% (95% CI: 88.2%-93.7%), 90.7% (95% CI: 86.9%-94.5%), and 96.6% (95% CI: 93.4%-99.9%), respectively, while the 5-year OS were 96.9% (95% CI: 95.3%-98.6%), 98.2% (95% CI: 96.5%-100%), and 98.3% (95% CI: 96.0%-100%), respectively. Notably, no statistically significant differences were observed in either DFS or OS among these three groups, with P-values of 0.10 and 0.77, respectively (**Figures 2A**, **3A**).

In comparison to the US+/MG- group, the US+/MG+ group exhibited no statistically significant differences in either disease-free survival (DFS) or overall survival (OS) at the baseline level (P>0.05). Upon adjusting for factors such as patient age, pathological status, and treatment status, this lack of significant difference persisted for both DFS and OS (P>0.05), as evident in **Table 3**, **4**.

**Table 3:** Hazards ratios for breast cancer disease-free survival of US+/MG-, US+/MG+ and MG+/US- NPBC.

NPBC	US+/MG-	US+/MG+	MG+/US-
Number	419	225	118
Number of Event	38	22	4
^&^HR (95%CI)	1.00	1.083(0.641-1.832)	0.364(0.129-1.019)
*^&^P*		0.764	0.054
^^^HR (95%CI)	1.00	0.952(0.556-1.629)	0.293(0.101-0.851)
*^^^P*		0.858	0.023*
^†^HR (95%CI)	1.00	0.979(0.569-1.684)	0.339(0.118-0.979)
*^†^P*		0.939	0.045*
^‡^HR (95%CI)	1.00	1.109(0.652-1.889)	0.406(0.144-1.150)
*^‡^P*		0.701	0.089
^&^HR (95%CI)	1.00	1.134(0.666-1.930)	0.324(0.114-0.924)
*^&^P*		0.643	0.035*
^$^HR (95%CI)	1.00	0.981(0.566-1.701)	0.288(0.098-0.849)
*^$^P*		0.945	0.023*

^&^HR Unadjusted hazard ratio.

^^^HR hazard ratio and P value adjusted for age and stage.

^†^HR hazard ratio and P value adjusted for stage, grade and pathological substype.

^‡^HR hazard ratio and P value adjusted for age, grade and pathological substype.

^&^HR hazard ratio and P value adjusted for age, surgery and postoperative treatment.

^$^HR hazard ratio and P value adjusted for age, stage, grade, pathological subtype, surgery and postoperative treatment.

**Table 4:** Hazards ratios for breast cancer overall survival of US+/MG-, US+/MG+ and MG+/US- NPBC.

NPBC	US+/MG-	US+/MG+	MG+/US-
Number	419	225	118
Number of Event	19	11	4
^*^HR (95%CI)	1.00	1.047(0.498-2.201)	0.699(0.238-2.056)
^*^P		0.903	0.516
^^^HR (95%CI)	1.00	1.012(0.476-2.151)	0.779(0.252-2.414)
*^^^P*		0.975	0.666
^†^HR (95%CI)	1.00	1.155(0.534-2.499)	1.015(0.327-3.147)
*^†^P*		0.714	0.980
^‡^HR (95%CI)	1.00	1.244(0.579-2.667)	1.057(0.344-3.255)
*^‡^P*		0.575	0.922
^&^HR (95%CI)	1.00	1.199(0.564-2.549)	0.688(0.228-2.075)
*^&^P*		0.636	0.507
^$^HR (95%CI)	1.00	1.182(0.537-2.597)	0.968(0.305-3.071)
*^$^P*		0.678	0.956

^*^HR Unadjusted hazard ratio.

^^^HR hazard ratio and P value adjusted for age and stage.

^†^HR hazard ratio and P value adjusted for stage, grade and pathological substype.

^‡^HR hazard ratio and P value adjusted for age, grade and pathological substype.

^&^HR hazard ratio and P value adjusted for surgery and postoperative treatment.

^$^HR hazard ratio and P value adjusted for age, stage, grade, pathological subtype, surgery and postoperative treatment.

The MG+/US- and US+/MG- groups did not significantly differ in terms of either disease-free survival (DFS) or overall survival (OS) (P>0.05). However, upon adjusting for age and tumor stage, the MG+/US- group exhibited a superior DFS, with a hazard ratio (HR) of 0.293 (95% CI: 0.101-0.851, P = 0.023), indicating a reduced risk of disease recurrence. This advantage persisted even after further adjustments for tumor stage, histological grading, and pathological classification of breast cancer, where the MG+/US- group still demonstrated a better DFS (HR 0.339, 95% CI: 0.118-0.979, P = 0.045). Consistent findings were observed regardless of whether adjustments were made for age and treatment alone or in combination with tumor stage, classification, and treatment, reinforcing the notion of improved DFS in the MG+/US- group. However, when adjustments encompassed age, histological grade, and pathological classification of breast cancer, the MG+/US- group did not show better DFS. In contrast, no significant difference in OS was detected between the MG+/US- and US+/MG- groups, regardless of the adjustments made for patient age, pathological status, and treatment status (**Table 4**). These findings highlight the potential clinical implications of considering the MG+/US- group in the management of breast cancer patients.

Upon comparing survival outcomes between the MG^+^/US^-^ group and the US^+^/Any MG group, our analysis demonstrated a significant improvement in disease-free survival (DFS) in the MG^+^/US^-^ cohort (P = 0.035; **Figure 2B**), whereas no statistically significant difference was observed in overall survival (OS) between the groups (P = 0.48; **Figure 3B**). Further evaluation using the Cox proportional hazards model confirmed the superior DFS in the MG^+^/US^-^ group, with an unadjusted hazard ratio (HR) of 0.353 (95% CI: 0.128–0.972; P = 0.044). This advantage in DFS became more pronounced after adjustment for age and tumor stage, yielding an adjusted HR of 0.299 (95% CI: 0.106–0.848; P = 0.023). Subsequent adjustments for additional covariates—including staging, tumor grade, and molecular subtype—attenuated the statistical significance of the DFS difference in some models, as detailed in **Supplementary Table 2**. Notably, however, DFS remained significantly improved after controlling for age and postoperative treatment. In contrast, OS consistently showed no significant difference across all adjusted and unadjusted models.

There were no statistically significant differences observed in either disease-free survival (DFS) or overall survival (OS) when comparing the US+/MG+ group with the anyUS/MG+ group (as depicted in **Figures 2C**, **3C**), nor were there any significant variations in DFS or OS between the US+ or MG+ group and the group where both US and MG were positive (as illustrated in **Figure 2D**, **3D**).

In univariate analysis, we identified several significant prognostic factors influencing DFS in NPBC. These include age at diagnosis, lymph node status, TNM stage, tumor histological grade, LVI, ER status, Hormone receptor status, breast cancer subtypes, surgical intervention, chemotherapy, and endocrine therapy (**Supplementary Figure 2**, P<0.05). Similarly, for overall survival (OS), we observed that TNM stages of BC, LVI, ER status, Hormone status, pathological subtype, and surgical intervention emerged as prognostic indicators (**Supplementary Figure 3**, P<0.05).

Following the results of our univariate analysis, we delved deeper into the prognostic factors of NBPC through a multivariate analysis. Prior to this, we conducted a multicollinearity test to ensure the independence of the variables, excluding those with Variance Inflation Factors (VIFs) exceeding 4. In the multivariate analysis for DFS, seven key factors emerged: age, tumor stage, tumor histological grade, LVI, estrogen receptor (ER) status, surgical intervention, and chemotherapy. When compared to patients under 40, those aged 50–59 demonstrated a better DFS (HR 0.427, 95% CI: 0.192-0.949, P = 0.037). Conversely, patients with stage IIIa and IIIb tumors had a poorer prognosis (P = 0.014 and P = 0.011, respectively), and the presence of LVI significantly worsened DFS (HR 2.413, 95% CI: 1.101-5.285, P = 0.028). ER-positive patients fared better, with a lower risk of DFS events (HR 0.437, 95% CI: 0.235-0.814, P = 0.009) (**Supplementary Figure 4**). In the multivariate analysis for OS, tumor stage, LVI, ER status, surgical approach, and chemotherapy status were included. However, only ER status emerged as a significant predictor of OS, with ER-positive patients exhibiting a more favorable prognosis (HR 0.301, 95% CI: 0.141-0.632, P = 0.002) (**Supplementary Figure 5**).

We further refined our understanding of prognostic factors by performing a subgroup analysis, comparing the US+/AnyMG group with the US-/MG+ group. Among patients with LVI negative, a notable difference in disease-free survival (DFS) was observed, with the US-/MG+ group exhibiting a significantly better DFS, as evidenced by an HR of 0.311 (95% CI: 0.097-0.996, P = 0.049). Additionally, within the subgroup of patients who underwent breast-conserving surgery, the US-/MG+ group continued to demonstrate a superior DFS, with an HR of 0.293 (95% CI: 0.09-0.952, P = 0.041). Furthermore, in the subset of patients who did not receive anti-HER2 therapy, the DFS of the US-/MG+ group surpassed that of the US+/AnyMG group (**Supplementary Figure 6**).

## 4 Discussion

Breast cancer stands as the most prevalent malignant tumor affecting women worldwide, with its incidence rate on a steady rise, juxtaposed against a decline in mortality rates over the past few decades (18).Emphasizing the paramount importance of early detection and timely treatment in mitigating breast cancer mortality, imaging modalities such as Ultrasound (US) and Mammography (MG) play pivotal roles in screening for this disease at its nascent stages, thereby contributing to reduced mortality (19). Our research underscores this by revealing a 5-year DFS of 91.7% (95% CI: 89.8%-93.7%) and a 5-year OS of 97.5% (95% CI: 96.4%-98.6%) among patients with non-palpable breast cancer detections. These promising outcomes underscore the vital necessity and profound impact of screening programs in fostering favorable prognoses for breast cancer patients.

As early diagnosis remains paramount, breast cancer screening initiatives have proliferated globally (20). Nevertheless, amidst this expansive pursuit, the concern of overdiagnosis must be reckoned with. Prior research has highlighted this issue, with notable studies illustrating its prevalence. Bleyer A et al. documented a substantial escalation in the incidence of ductal carcinoma *in situ* (DCIS) among women over 50 in the United States, from 10 per 100,000 in the 1980s to approximately 90 per 100,000 by the dawn of the 21st century, underscoring the potential for overdiagnosis given that only 8 out of 122 early-detected cancers are projected to progress to advanced stages (21). Moreover, Zahl PH et al.’s comprehensive comparative analysis of breast cancer incidence rates in Swedish counties, spanning the years 1986 to 1990, prior to and subsequent to mammography (MG) screening, revealed a striking augmentation in the 4-year cumulative incidence rate among the screened cohort, reaching 982 cases per 100,000 individuals, compared to 658 cases per 100,000 in the control group. This disparity translates to a relative risk of 1.49, with a 95% confidence interval ranging from 1.41 to 1.58, underscoring the significant impact of mammography screening. Notably, despite prevalence screening in the control group, the 6-year cumulative incidence rate of invasive breast cancer continued to be elevated in the screened population, with a rate of 1443 per 100,000 versus 1269 per 100,000 in the control group, yielding a relative risk of 1.14 (95% CI 1.10-1.18). This observation implies that numerous invasive breast cancers initially detected through repeated mammography screening may not persist to be identified by subsequent screenings over a 6-year period, suggesting that the natural trajectory of many of these screen-detected cancers is towards spontaneous regression (22). Christian et al. reported a concerning trend in Norway’s breast cancer screening program, where the ratio of overdiagnosed cancers per breakthrough cancer death prevented escalated from 3.2 in 1996 to 5.4 in 2016. This escalation was more pronounced when considering varying degrees of mobility reduction, with the ratio climbing from 7.4 to 14.0 for an 8.7% reduction and from 12.8 to 25.2 for a 5% reduction (23). Furthermore, more research underscores the potential for spontaneous regression in some screen-detected breast cancers, as evidenced by Zackrisson S et al.’s 10-year trial, which detected 741 cases in the screened group and 591 in the control group. Over the subsequent 15 years, the difference between the two groups narrowed significantly, from 150 to 115 cases, indicative of overdiagnosis (24).Welch HG et al. estimated the risk of overdiagnosis associated with mammography-detected cancers to be approximately 24%, emphasizing the need for a balanced approach in screening practices (25). In our study, mammography (MG) examination revealed a heightened detection of intraductal cancers, with a notable discrepancy among three groups. Specifically, the proportion of intraductal cancers among breast cancer cases detected solely by mammography (US-/MG+ group) exceeded 50%, contrasting sharply with the 13.4% observed in the US+/MG- group and the 17.8% in the US+/MG+ group. This significant statistical difference underscores a potential concern regarding overdiagnosis in mammography screening. Further analysis of survival outcomes revealed that the disease-free survival (DFS) of patients in the US-/MG+ group was markedly superior to that of the US+ group, suggesting a potentially more favorable prognosis for this subgroup of NPBC patients detected through screening. While the OS curve of these patients demonstrated a trend towards benefit, statistical testing failed to yield a significant difference, possibly due to the inherently “low-risk” nature of NPBC, rendering OS differences less pronounced. It is conceivable that a more pronounced OS difference may emerge with an expanded sample size or extended follow-up period.

In our prior research endeavors, we conducted a multi-center, randomized controlled trial to ascertain whether US would emerge as a superior imaging modality for breast cancer screening among high-risk Chinese women (9). Our findings revealed that US demonstrated greater sensitivity than MG (100.0% versus 57.1%, P = 0.04), accompanied by enhanced diagnostic accuracy (0.999 versus 0.766, P = 0.01), while maintaining comparable specificity (100% versus 99.9%, P = 0.51). Notably, the cost of US was substantially lower than MG, ranging from $20 to $30 compared to $65 to $75 (9). Additionally, US being radiation-free allows for repeated examinations and is more favorably accepted by patients. According to the guidelines of the Chinese Anti-Cancer Association, both MG and US are recommended for breast cancer screening (26). Our study further observed that the US+/MG+ NPBC group exhibited a superior PPV of 34.7%, whereas the US+/MG- group had a PPV of 21.6%, and the US-/MG+ group registered a PPV of 22.6%. Notably, US detected a higher proportion of invasive carcinomas, exceeding 80% in both US+ subgroups. Outside China, although population-based mammographic screening has been implemented for over two decades in several Asian countries, its benefits for women in their 40s remain limited. Given that mammography exhibits reduced sensitivity due to masking effects, ultrasound (US) has been proposed as a potential adjunctive screening tool in many Asian countries (27). Supplemental screening breast ultrasound may benefit women with dense breast tissue, elevated breast cancer risk, or a personal history of breast cancer (28). A secondary analysis of data from the J-START study conducted in Japan suggested that the addition of ultrasound improved sensitivity in both dense and non-dense breasts and detected more early-stage and invasive cancers among asymptomatic women aged 40–49 years (29). In the Western countries, US is frequently employed as an adjunct to MG, particularly for breasts with >50% density (11, 12). The ACRIN 6666 study underscores that US identifies more invasive and node-negative cancers, whereas MG tends to detect more ductal carcinoma *in situ* (DCIS) (P < 0.001) (13). These findings are consistent with the results observed in the present study. Researchers from the ACRIN 6666 study also emphasized that invasive cancers undetected by mammography may manifest as interval cancers with poorer prognosis, implying that the detection of asymptomatic, mammographically occult, node-negative invasive carcinomas through US could potentially reduce breast cancer mortality (11).Regarding survival outcomes, the US-/MG+ NPBC group demonstrated superior DFS compared to the US+/AnyMG group, with a trend towards improved OS, albeit not statistically significant. This suggests that US-/MG+ NPBC may represent an “ultra-low-risk” subtype of breast cancer, characterized by a higher prevalence of DCIS and better prognosis.

In our present study, we observed no statistically significant variations among the three subgroups of NPBC concerning age, lymph node status, hormone receptor status, Ki67 index, receipt of endocrine therapy, chemotherapy, or targeted therapy. Remarkably, MG demonstrated a heightened capacity to identify multifocal cancers (p=0.003), potentially attributed to its superior sensitivity towards multifocal and multicentric lesions (30). Additionally, patients within the US-/MG+ cohort underwent significantly more breast-conserving surgeries (p<0.001) and radiotherapy(p<0.001), aligning with Braun B et al.’s findings (31). The latter reported that individuals with mammography-screening-program-targeted mammographically screened populations (MSP) detected by MG underwent breast-conserving surgery more frequently (75% versus 62%). This phenomenon may be explained by MG’s proficiency in detecting DCIS and NPBC in earlier stages, thereby facilitating more breast-conserving surgeries.

In analyzing survival outcomes, the 5-year DFS among all NPBC patients stood at an impressive 91.7% (95% CI 89.8%-93.7%). Similarly, the 5-year OS reached 97.5% (95% CI 96.4%-98.6%). These results suggest that NPBC identified through screening represents a “low-risk” subtype of breast cancer, portending a favorable prognosis. Notably, NPBC cases exhibiting positive MG findings without corresponding malignant US findings exhibited an even better prognosis. Precisely, the DFS in the MG+/US- subgroup significantly surpassed that of the US+/AnyMG subgroup(P = 0.035). Other studies have also suggested that 60–70% of MG-detected breast cancers were DCIS (14, 15). A Finnish study further underscores the prognostic value of mammography screening, establishing it as an independent predictor of enhanced survival outcomes in breast cancer (P<0.0001) (32). Additionally, a Netherlands investigation has demonstrated that, in comparison to interval cancers, cancers detected via screening harbor a lower risk of aggressive tumor biology, with 68% classified as low risk, and a notable 54% falling into the ultra-low risk category (p=0.001). This translates to an odds ratio (OR) of 2.33, indicating a significant advantage for screened cancers (p<0.0001; 95% CI: 1.73-3.15) (33). Furthermore, MG-detected breast mass/nodule is usually (if not always) positive to US, and macro-calcification alone is almost always considered benign (16). Calcium deposition, a frequent benign finding in asymptomatic ductal carcinoma *in situ* (DCIS) and early-stage cancers, is highly detectable through X-ray-based modalities, underscoring their sensitivity in identifying such deposits (14).These micro-calcifications are often reported in high numbers in autopsy studies, indicating that people might not detect the lesion until death (34).On the contrary, ultrasound has demonstrated significant potential in detecting microcalcifications that are accompanied by palpable lumps, nodules, or notable architectural distortions of breast tissue. This modality provides a valuable addition to MG screening, allowing for a more comprehensive assessment of breast lesions and avoiding unnecessary breast biopsy (35, 36). The Mayo BBD cohort study highlights an increased breast cancer risk among women with benign breast disease and biopsy history, stratified by the extent of epithelial abnormalities (16).Consequently, we posit that mammography-positive yet ultrasound-negative non-palpable breast cancers (MG+/US- NPBC) should be categorized as “ultra-low risk” BC. For microcalcifications detected solely through molybdenum target screening without ultrasound corroboration, the likelihood of invasive cancer is approximately 10%, reinforcing the efficacy and safety of using ultrasound as a risk-stratification adjunct to MG based screening.

We have performed a thorough examination of prognostic factors in NPBC utilizing both univariate and multivariate Cox regression analyses. Our findings underscore the significance of age, TNM stage, LVI, and ER status as independent risk factors for DFS among patients. While age, TNM stage, and ER status are well-established prognostic indicators in breast cancer, corroborated by numerous prior studies. It is noteworthy that the presence of LVI serves as an independent risk factor for disease-free survival in NPBC, conferring significant clinical implications for identifying patients at high risk of recurrence and deserving wider clinical attention. A comprehensive cohort study has demonstrated that the presence of LVI is intimately linked to inferior outcomes, manifested by shortened breast cancer-specific survival (BCSS) and distant metastasis-free survival (DMFS), particularly among the low-risk pT1-pT2/N0 subgroup. Notably, LVI emerges as a robust high-risk criterion, sufficient to reclassify patients into the high-risk group, conferring a risk level comparable to that associated with 1 to 3 positive lymph nodes (pN1) or a change in tumor size category (from pT1 to pT2) (37). Reinforcing these findings, a study conducted in the Netherlands underscores LVI’s independent prognostic value for BCSS in young, lymph node-negative triple-negative breast cancer (TNBC) patients (38).Additionally, another investigation has highlighted LVI’s significant association with prognosis in hormone receptor-positive, N1 breast cancer patients (39).In the esteemed NCCN guidelines, a recommendation stands that patients diagnosed with T2 tumors accompanied by LVI should undergo WBRT with or without a boost to the tumor bed, in conjunction with regional nodal irradiation (RNI) radiotherapy. While this underscores the pivotal role of LVI in guiding radiotherapy decisions, it is noteworthy that LVI’s influence has yet to be fully elucidated in the context of other crucial adjuvant therapies, particularly chemotherapy (40). Consequently, we assert that LVI deserves heightened consideration and scrutiny in the intricate process of formulating comprehensive treatment strategies, ensuring that patients receive tailored interventions that effectively address their unique tumor characteristics and prognosis.

The status of estrogen receptor (ER) emerges as an independent prognostic indicator for overall survival in NPBC patients, with ER-positive NPBC patients exhibiting significantly better overall survival (P = 0.002), aligning with previous research endeavors. In the univariate analysis conducted, several factors including TNM stage, LVI, surgery, and chemotherapy administration were identified as potential contributors to overall survival in non-parous breast cancer (NPBC) patients. Nevertheless, upon conducting a multivariate analysis, no statistically significant differences were discerned in the influence of these factors on overall survival outcomes. This suggests that, when considered collectively, these variables do not independently confer a significant impact on survival prognosis in NPBC. This lack of significance may stem from the inherently favorable overall survival rates observed in NPBC patients, where the number of overall survival (OS) events did not reach the threshold necessary to establish statistical distinction.

Our subgroup analysis meticulously examined two distinct patient groups: MG+/US- and US+/AnyMG. Our key findings highlighted that, among patients who were LVI-negative, had undergone breast-conserving surgery, and had not received anti-HER2 treatment, the MG+/US- cohort demonstrated superior DFS rates in comparison to their US+ counterparts. This observation may be attributed to the relatively indolent biological behavior of MG^+^/US^-^ BC, which are often diagnosed as DCIS or low-grade invasive carcinomas, typically exhibiting lower proliferative activity and a higher prevalence of Luminal subtype molecular profiles. These characteristics provide a biological basis for their favorable prognosis. From a clinical perspective, these findings support the adoption of a more individualized treatment strategy for MG^+^/US^-^ BC. After thorough multidisciplinary evaluation and informed consent, de-escalation of treatment intensity—such as reducing the extent of surgical resection or omitting certain adjuvant therapies—may be considered for selected low-risk patients. However, it is important to note that accurate preoperative determination of LVI status and HER2 expression remains challenging. Therefore, surgical pathology findings should continue to serve as the primary basis for final treatment decisions. Future studies should further explore the correlation between non-invasive imaging biomarkers and tumor biology to facilitate the development of a more precise preoperative risk assessment system. On the other hand, comprehensive preoperative evaluation incorporating US, mammography, and MR imaging provides valuable information regarding the feasibility of breast-conserving surgery. Among patients deemed eligible for this approach, those with MG^+^/US^-^ BC are likely to have a more favorable prognosis. In other words, MG^+^/US^-^ BC patients who qualify for breast-conserving surgery may be considered to have a lower-risk disease profile.

The findings of this study provide a theoretical basis for further exploration of a “watch-and-wait” strategy in patients with MG^+^/US^-^ NPBC. Given the exceptionally favorable DFS and OS observed in this subgroup, active surveillance—rather than immediate surgical intervention—may represent a feasible clinical alternative for a carefully selected subset of low-risk patients, such as those of advanced age, with significant comorbidities, or who strongly decline surgery. However, the implementation of such a strategy must be underpinned by a multidisciplinary evaluation and thorough informed consent, ensuring that patients fully understand the potential risks associated with deferred surgery. A stringent follow-up protocol should be established, including semiannual breast ultrasound and mammography, supplemented with breast MRI when necessary. Image-guided core needle biopsy is also recommended to obtain a definitive pathological diagnosis prior to considering surveillance. Furthermore, clear criteria for surgical intervention must be defined in advance to address either disease progression or a change in patient preference. Although limited existing evidence suggests that active surveillance may be a viable option for certain patients with MG^+^/US^-^ NPBC, its long-term safety and efficacy require further validation through well-designed prospective studies.

From a biological mechanism perspective, mammary microcalcifications are primarily composed of calcium oxalate or hydroxyapatite crystals, which are associated with benign and malignant breast lesions, respectively. Microcalcifications that are visible on mammography but often undetectable by ultrasound may be related to benign conditions such as secretory disease or fat necrosis, yet they can also represent the earliest sign of malignant breast disease. In the context of breast cancer, studies have revealed that microcalcifications can be actively produced by cells undergoing epithelial-mesenchymal transition (EMT) and acquiring a mesenchymal phenotype. Under stimulation by bone morphogenetic protein-2 (BMP-2), these cells may further adopt an osteoblast-like phenotype, becoming breast osteoblast-like cells (BOLCs), which demonstrate the ability to synthesize and secrete hydroxyapatite (HA) microcalcifications. The overexpression of ER in breast cancer may participate in this tumor cell transdifferentiation toward an osteoblastic lineage, serving as a key regulator in osteogenic differentiation and function (41). Additionally, some studies have indicated that microcalcification-associated breast tumors exhibit a higher frequency of cases positive for both estrogen and progesterone receptors, which is generally considered a favorable prognostic indicator (42). Nevertheless, the potential role of microcalcifications and their interplay with the breast microenvironment in the early development and progression of breast cancer remains inadequately elucidated (43).

This study acknowledges several limitations that are essential to consider. First, regarding study design and data representativeness, this research is a single-center retrospective analysis, and the sample size is relatively limited due to constraints in the accumulation of clinical data from a single institution. The single-center design may result in data that primarily reflect the clinicopathological characteristics (such as tumor stage, distribution of pathological types, and expression of molecular markers) and prognostic patterns of breast cancer patients treated at this institution. Differences among medical centers—including patient inclusion criteria (e.g., whether there is a tendency to enroll high-risk populations), treatment protocols (e.g., selection of surgical methods and adjuvant therapy strategies), and follow-up management practices (e.g., follow-up intervals and detection methods)—may limit the external validity of our findings. Moreover, as our institution is a tertiary academic hospital located in an urban area, the study cohort may not fully represent patients from rural regions, who may differ in terms of socioeconomic status, health awareness, access to specialized care, and stage at diagnosis. Therefore, caution is warranted when generalizing these results to other populations or healthcare settings, particularly non-Chinese populations. Furthermore, as a retrospective study, this research relies on the extraction and analysis of historical medical records. Although we implemented strict case selection and cross-verified key variables to minimize bias, there remains the possibility of incomplete documentation of certain critical clinical information. Such gaps may include detailed patient comorbidity histories, specific postoperative rehabilitation interventions, or minimal residual disease detection results. These limitations could potentially affect the comprehensiveness of data analysis and the accuracy of the conclusions. Second, this study is subject to potential risk of outcome underestimation. As a retrospective analysis, it lacks the capacity for real-time monitoring throughout the patients’ diagnostic and treatment courses, which may introduce bias in long-term follow-up metrics—such as delayed detection of micro-metastases, loss to follow-up, or incomplete medical documentation. Furthermore, given the clinical nature of non-palpable breast cancer (NPBC), which is characterized by occult presentation and relatively indolent progression, certain subclinical recurrence or metastasis events (e.g., bone or brain metastases) may not become apparent within a relatively short follow-up period. This could lead to an underestimation of the true disease risk in this patient population. Furthermore, in the key subgroup analysis, the relatively small sample size of the MG^+^/US^-^ subgroup significantly limited the statistical power of this segment of the study. As a candidate “ultra-low-risk” subtype of primary interest, the limited sample size of MG^+^/US^-^ NPBC introduced several methodological constraints. The inadequate number of cases hindered more granular stratified analyses—such as further categorization by tumor size, histologic grade, or molecular subtype—thereby preventing a detailed exploration of prognostic heterogeneity across different clinical and pathological features. Moreover, the precision of estimating key outcome measures, including disease-free survival and hazard ratios for recurrence, was compromised, as reflected in wider 95% confidence intervals. This not only reduces the reliability of the estimates but may also obscure clinically meaningful effects. Additionally, owing to insufficient statistical power, it was challenging to robustly compare survival outcomes between this subgroup and other subgroups, which consequently weakens the strength of evidence supporting the hypothesis that MG^+^/US^-^ NPBC represents an ultra-low-risk population. Finally, from the perspective of follow-up duration, although this study employed a 5-year follow-up period to evaluate patient survival outcomes and obtained informative 5-year disease-free and overall survival rates, this timeframe remains insufficient to fully capture the long-term prognosis of NPBC patients. Given the indolent nature and generally favorable prognosis of NPBC, the 5-year follow-up may not adequately reflect late recurrence or metastasis events occurring beyond this period. Moreover, for low-risk subtypes such as MG^+^/US^-^ NPBC, the long-term recurrence patterns and metastatic risks may differ from those of other subtypes, and short-term follow-up is unable to fully elucidate these characteristics. Therefore, extended prospective follow-up spanning 10 years or more is warranted to more accurately delineate the long-term recurrence dynamics, metastatic risks, and overall survival trajectories in NPBC patients. Such efforts would provide stronger evidence to guide the formulation of precision follow-up strategies and treatment plans.

## 5 Conclusion

The findings suggest that MG+/US- NPBC exhibits a favorable prognosis, characterized by a higher prevalence of DCIS, potentially positioning it as an ‘ultra-low risk’ cancer subtype. This underscores the potential of US in refining the risk stratification of screen-detected NPBC into two categories: regular low risk (comprising both US+/MG+ and US+/MG- subgroups) and the aforementioned ultra-low risk (MG+/US-). By leveraging this stratification, more tailored management strategies can be devised to optimize patient care and outcomes.

## Data Availability

The raw data supporting the conclusions of this article will be made available by the authors, without undue reservation.
