# Supplementary material for: Risk stratification by ultrasound and mammography for screen-detected non-palpable breast cancer in Chinese women
Source: Front Oncol. 2025 Oct 17;15:1555743. doi: 10.3389/fonc.2025.1555743 (PMC12575149; doi:10.3389/fonc.2025.1555743)
Supplement: Supplementary file 1 [file DataSheet1.docx]

Supplementary Materials


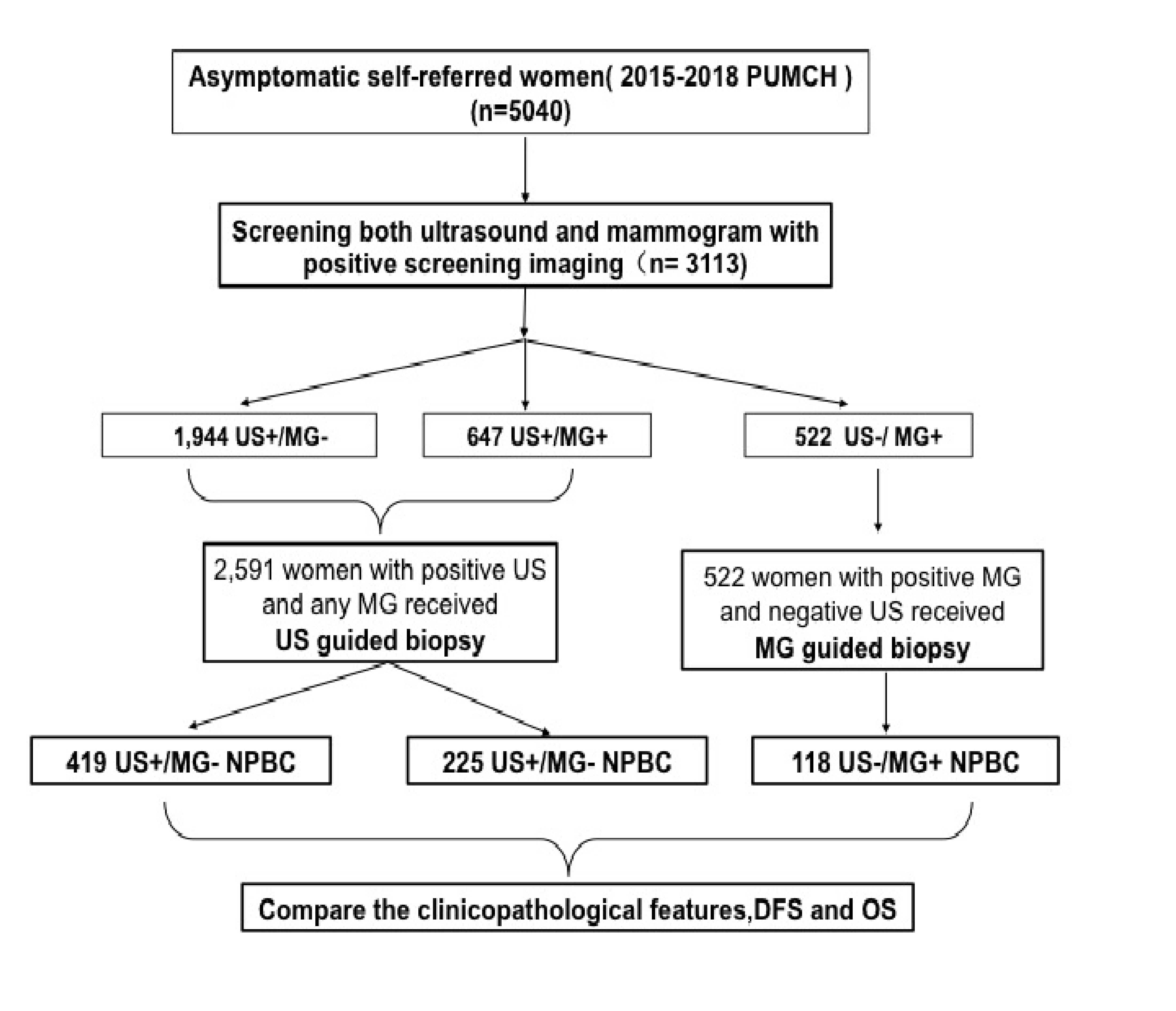


Supplementary Figure 1. Diagram of the research design.


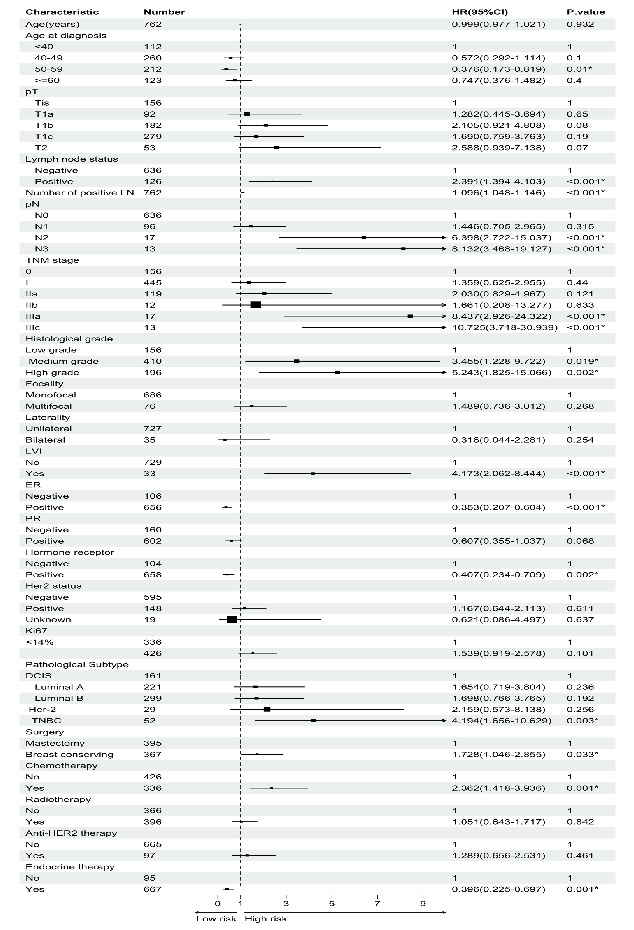


Supplementary Figure 2. Univariate analysis of DFS related prognostic factors of NPBC patients. The DFS prognostic factors for these three groups of NPBC included different age groups at diagnosis, lymph node status, pN, TNM stage, Histological grade, LVI, ER, HR, Pathological Subtype, Surgery, chemotherapy and endocrine therapy (P<0.05).


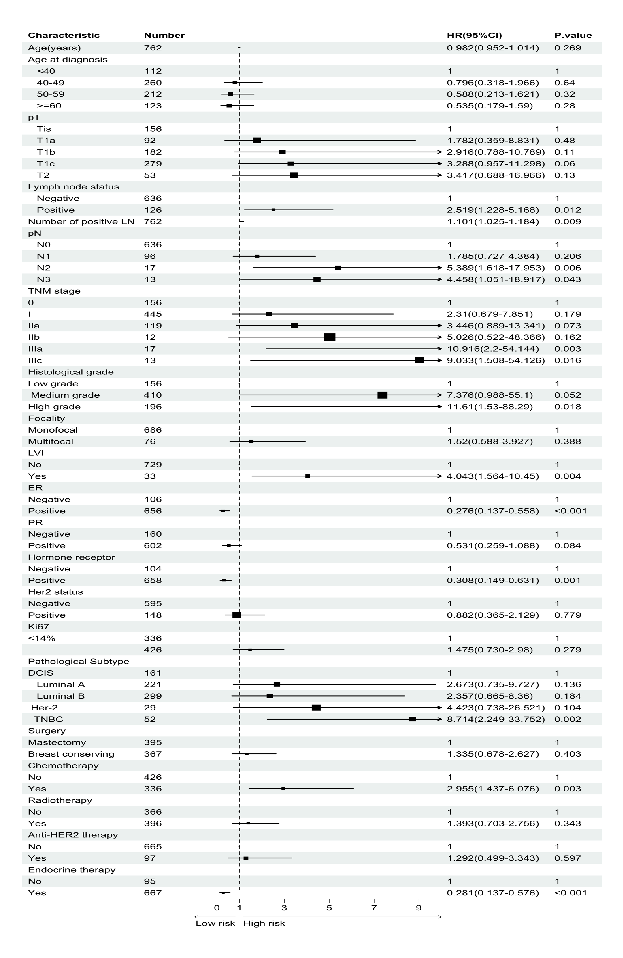


Supplementary Figure 3. Univariate analysis of OS related prognostic factors of NPBC patients. The OS prognostic factors for these three groups of NPBC included different TNM stage, LVI, ER, HR, Pathological Subtype, Surgery and(P<0.05).


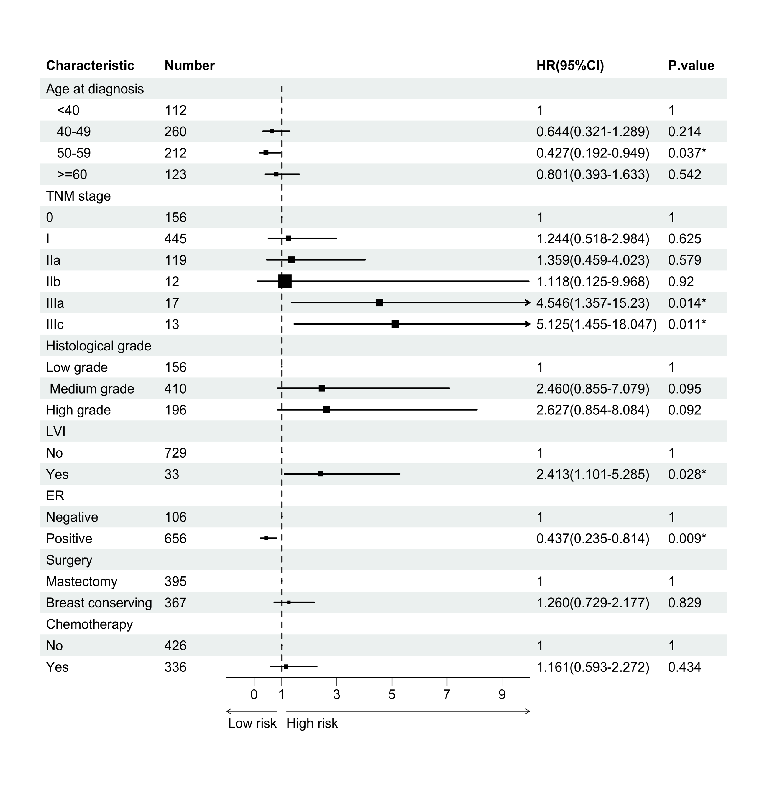


Supplementary Figure 4. Multivariate analysis of DFS related prognostic factors of NPBC patients. The DFS prognostic factors for these three groups of NPBC included different age groups at diagnosis, TNM stage, LVI and ER (P<0.05).


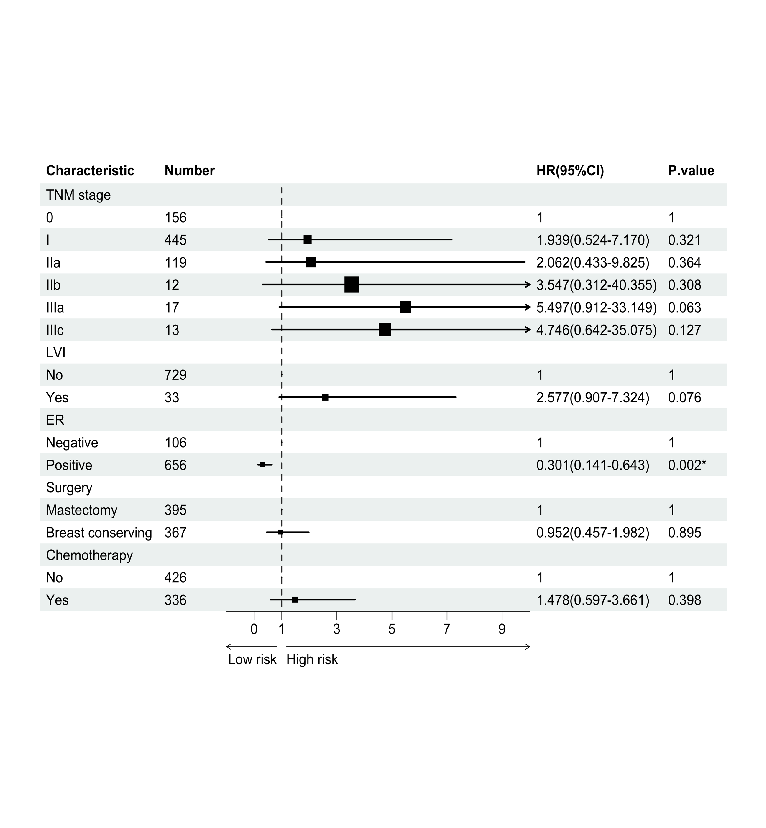


Supplementary Figure 5. Multivariate analysis of OS related prognostic factors of NPBC patients. ER was the OS prognostic factor for these three groups of NPBC (P=0.002).


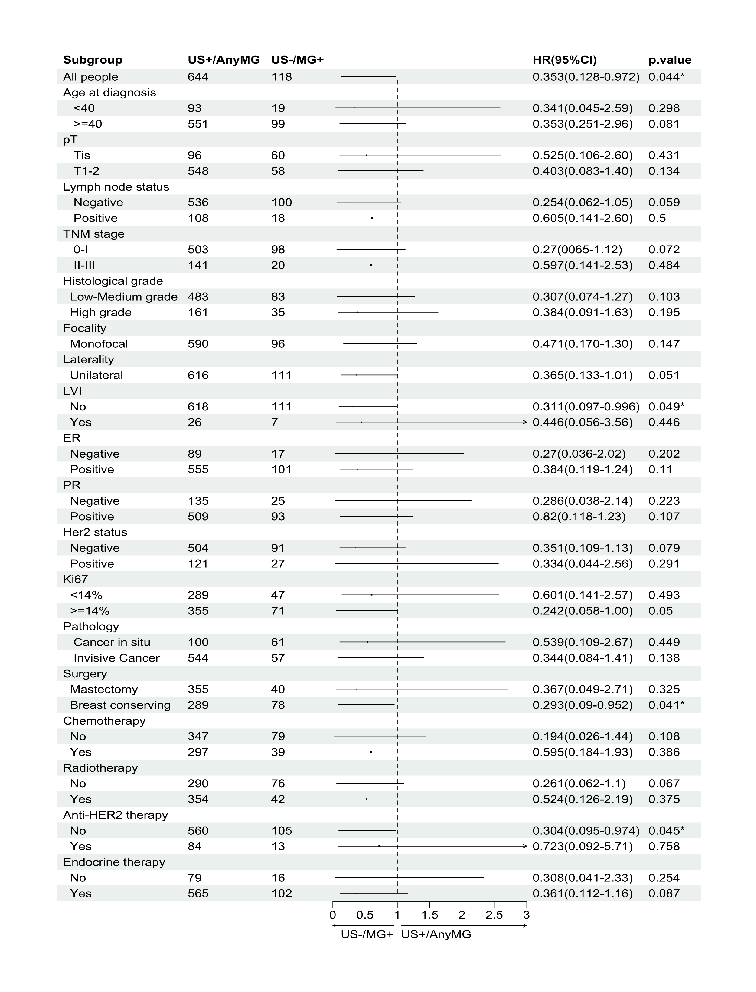


Supplementary Figure 6. Subgroup analyses by DFS related prognostic factors for US+/AnyMG or US-/MG+ groups: There was a significant difference in DFS between the two groups of LVI negative, breast conserving, and non-anti-HER2 therapy patients (P<0.05).

### **Supplementary Table 2.** Recurrence, metastasis and survival outcomes in the NPBC cohort

| **Variable** | **Number** | **Percentage (%)** |
| --- | --- | --- |
| Recurrence/Metastasis events |  |  |
| Any recurrence or metastasis | 64 | 2.06 |
| Local recurrence | 13 | 0.42 |
| Distant metastasis | 51 | 1.64 |
| Lung metastasis | 13 | 0.48 |
| Bone metastasis | 15 | 0.5 |
| Liver metastasis | 5 | 0.16 |
| Cervival lymph node metastasis | 8 | 0.26 |
| Multiple metastases (≥2 organs) | 10 | 0.32 |
| Survival outcomes |  |  |
| All-cause mortality | 34 | 1.09 |
| Breast cancer-related mortality | 27 | 0.87 |
| Non-breast cancer-related mortality | 7 | 0.22 |

**Supplementary Table 2.** Hazard Ratios for Breast Cancer Disease-Free Survival of US+/AnyMG and MG+/US- NPBC

| **NPBC** | **US+/AnyMG** | **MG+/US-** |
| --- | --- | --- |
| Number | 644 | 118 |
| Number of Event | 60 | 4 |
| ^&^HR (95%CI) | 1.00 | 0.353(0.128-0.972) |
| *^&^P* |  | 0.044* |
| ^^^HR (95%CI) | 1.00 | 0.299(0.106-0.848) |
| *^^^P* |  | 0.023* |
| ^†^HR (95%CI) | 1.00 | 0.342(0.121-0.965) |
| *^†^P* |  | 0.043* |
| ^‡^HR (95%CI) | 1.00 | 0.390(0.141-1.081) |
| *^‡^P* |  | 0.07 |
| ^※^HR (95%CI) | 1.00 | 0.309(0.111-0.864) |
| ^※^P |  | 0.025* |
| ^$^HR (95%CI) | 1.00 | 0.290(0.101-0.834) |
| ^$^P |  | 0.022* |

^&^HR Unadjusted hazard ratio.

^^^HR hazard ratio and P value adjusted for age and stage.

^†^HR hazard ratio and P value adjusted for stage, grade and pathological substype.

^‡^HR hazard ratio and P value adjusted for age, grade and pathological substype.

^※^HR hazard ratio and P value adjusted for age, surgery and postoperative treatment.

^$^HR hazard ratio and P value adjusted for age, stage, grade, pathological subtype, surgery and postoperative treatment.
